# Miscellany

**Published:** 1906-03

**Authors:** 


### MISCELLANY.

THE Warren Triennial Prize was founded by the late Dr. J. Mason Warren in memory of his father, and his will provides that the accumulated interest of the fund shall be awarded every three years to the best dissertation, considered worthy of a premium, on some subject in Physiology, Surgery, or Pathological Anatomy; the arbitrators being the physicians and Surgeons of the Massachusetts General Hospital.

The subject for competition for the year 1907 is on Some Special Subject in Physiology, Surgery, or Pathology. Dissertations must be legibly written, and must be suitably bound, so as to be easily handled. The name of the writer must be enclosed in a sealed envelope, on which must be written a motto corresponding with one on the accompanying dissertation. The amount of the prize for the year 1907 will be $500. In case no dissertation is considered sufficiently meritorious, no award will be made. Dissertations will be received until April 14, 1907. A high value will be placed on original work.

Herbert B. Howard,
Resident Physician, Massachusetts General Hospital.

---

POSITIONS OPEN.

The New York State Civil Service Commission will hold, at Albany, April 13 and 14, open competitive examinations for Medical Superintendent and First Assistant Physician at the New York State Hospital for the Treatment of Incipient Pulmonary Tuberculosis, at Raybrook in the Adirondacks. Examinations open to residents and non-residents of New York State. For particulars and application forms, address
Chief Examiner, State Civil Service Commission, Albany, N. Y.

